# Polymorphisms in DNA Repair and Xenobiotic Biotransformation Enzyme Genes and Lung Cancer Risk in Coal Mine Workers

**DOI:** 10.3390/life12020255

**Published:** 2022-02-09

**Authors:** Varvara Minina, Anna Timofeeva, Anastasya Torgunakova, Olga Soboleva, Marina Bakanova, Yana Savchenko, Elena Voronina, Andrey Glushkov, Alexander Prosekov, Aleksandra Fucic

**Affiliations:** 1 The Federal Research Center of Coal and Coal Chemistry of Siberian Branch, Federal State Budget Scientifc Institution, Russian Academy of Sciences, Department of Human Ecology, 650065 Kemerovo, Russia; vminina@mail.ru (V.M.); kotia1490@mail.ru (A.T.); soboleva.olga88@yandex.ru (O.S.); mari-bakano@yandex.ru (M.B.); yasavchenko@yandex.ru (Y.S.); ihe@list.ru (A.G.); 2 Department of Genetics and Fundamental Medicine, Kemerovo State University, 650000 Kemerovo, Russia; annateam86@gmail.com (A.T.); rector@kemsu.ru (A.P.); 3 Institute of Chemical Biology and Fundamental Medicine of SB RAS, Pharmacogenomics Laboratoriey, Lavrentiev Ave 8, 630090 Novosibirsk, Russia; voronina_l@mail.ru; 4 Institute for Medical Research and Occupational Health, 10000 Zagreb, Croatia

**Keywords:** lung cancer: miner, genetic polymorphism, *GSTM1* (deletion), *APEX1*, *XPD*, *NBS1*

## Abstract

Background: Currently coal mining employs over 7 million miners globally. This occupational setting is associated with exposure to dust particles, heavy metals, polycyclic aromatic hydrocarbons and radioactive radon, significantly increasing the risk of lung cancer (LC). The susceptibility for LC is modified by genetic variations in xenobiotic detoxification and DNA repair capacity. The aim of this study was to investigate the association between *GSTM1* (deletion), *APEX1* (*rs1130409*), *XPD* (*rs13181*) and *NBS1* (*rs1805794*) gene polymorphisms and LC risk in patients who worked in coal mines. Methods: The study included 639 residents of the coal region of Western Siberia (Kemerovo region, Russia): 395 underground miners and 244 healthy men who do not work in industrial enterprises. Genotyping was performed using real-time and allele-specific PCR. Results: The results show that polymorphisms of *APEX1* (recessive model: OR_adj_ = 1.87; CI 95%: 1.01–3.48) and *XPD* (log additive model: OR_adj_ = 2.25; CI 95%: 1.59–3.19) genes were associated with increased LC risk. *GSTM1* large deletion l was linked with decreased risk of LC formation (OR_adj_ = 0.59, CI 95%: 0.36–0.98). The multifactor dimensionality reduction method for 3-loci model of gene–gene interactions showed that the *GSTM1* (large deletion)*—APEX1* (*rs1130409*)*—XPD* (*rs13181*) model was related with a risk of LC development. Conclusions: The results of this study highlight an association between gene polymorphism combinations and LC risks in coal mine workers.

## 1. Introduction

Lung cancer is the leading neoplastic disease and the main cause of mortality among oncological patients worldwide [1]. Smoking has been confirmed as a key risk factor for LC cancer development, which increases its risk by more than five times [2]. Nevertheless, approximately 25% of LC patients are not smokers, [3], suggesting that the etiology of LC may also have a genetic and environmental origin. Air pollution (polycyclic aromatic hydrocarbons (PAHs)), dust, quartz nanoparticles, heavy metals and radiation are only some of the xenobiotics that may increase lung cancer risk [1]. Mixtures of these agents are characteristic of air pollution in coal mines [4]. All of them are genotoxic carcinogens and may contribute to a higher risk of neoplastic diseases in coal mine workers [5,6,7,8]. Many studies have shown that coal mine workers are more often affected by LC compared to subjects that are not affiliated with coal mining [9,10,11]. As it is estimated that over 44 million artisanal miners are currently employed across 80 countries [12], of which about 7 million are employed coal mining [13], it is of great significance to recognize risk factors and apply preventive measures.

Genome-wide association studies (GWAS) reported an association between LC risk and 45 loci, each having different significance and potential. The most significant were identified for SNPs located at the 15q25, 5p15 and 6p21 regions [14,15]. In combination with a transcriptome-wide association study (TWAS), it was shown that the gene most strongly associated with LC is IREB2. Additionally, a new lung adenocarcinoma susceptibility locus was revealed on 9p13.3 and associated with higher predicted expression of AQP3. IREB2 knockdown and AQP3 overproduction promote endogenous DNA damage. These findings indicate genes whose expression in lung tissue directly influences LC risk [16]. These results have been associated with LC risk in the case of specific types of exposure, especially radiation. Additionally to exposure to radon in the workplace, miners are also exposed to radon in their dwellings [17,18]. The measured radioactivity caused by radon may vary; for instance, in the coal mines of the Kuzbass region (Russian Federation), it ranges from 410 up to 6000 Bq/m^3^ [19].

The occupational setting in a coal mine is radiochemical. Thus, in addition to radiation, a significant component of LC risk is air pollution which contains PAH mixtures. Wang et al. (2020) identified a significant increase in the PAH metabolites 2-NAP, 2-FLU, 9-PHE, and 1-OHP in coal miners [20].

In coal mines, dust mostly contains PM2.5 and PM0.1 particles containing carbon, silicon dioxide, aluminum oxide, iron (III) oxide, sulfur oxide, calcium oxide, magnesium oxide, and titanium dioxide [21,22].

Glutation-S-transpherases, such as GSTM1, which takes part in the detoxification of potential carcinogens [23,24], similarly to DNA repair enzymes (APEX1, XPD and NBS1) [25,26,27], play a significant role in lung carcinogenesis. It is reported that the deletions of *GSTM1* and *GSTT1* genotypes are associated with a higher risk of LC, and that these subjects express a higher risk of LC for similar radon levels [28,29]. Additionally, both polymorphisms in genes involved in DNA repair and carriers of *GSTM1* deletion have an increased risk of LC in never-smokers exposed to radon [30,31].

APEX nuclease 1 (APEX1) is the enzyme responsible for the recognition and incision of apurinic/apyrimidinic (AP) sites after DNA damage caused by radiation, and it is an important redox modulator [32]. *APEX 1* variant rs1130409 has been shown to be associated with increased LC risk [26], but there is no information on its impact in case of exposure to ionizing radiation.

DNA repair gene *XPD* variant rs13181 was shown to contribute to the efficacy and toxicity of radiotherapy in patients with LC [33]. Caucasian populations show the most significant association between *XPD* rs13181 and LC risk [34].

A variant of Nijmegen breakage syndrome 1 *NBS1 rs1805794* was found to be associated with p53 mutations in LC; however, further investigations are required [35,36]. It is interesting that this variant has sex-specific activity and, in the case of cigarette smoke, increases the risk for LC only in males [27].

Increased risk of LC for carriers of *APEX1* variant rs1130409, *NBS1* (rs1805794) and *XPD* rs13181 was already described based on and associated with residential exposure to increased radon levels [37]. The current study is the first to investigate the association between *GSTM1* (large deletion), *APEX1* (*rs1130409*), *XPD* (*rs13181*) and *NBS1* (*rs1805794*) gene polymorphisms and LC risk in patients who worked as miners in coal mines exposed to radon and dust.

## 2. Materials and Methods

### 2.1. Participants and Blood Sample Collection

In the current study, 395 miners who worked in coal mines (Kuzbass, Western Siberia, Russian Federation) were examined. Subjects comprised 208 males diagnosed with LC treated at Kemerovo regional oncological center (Kemerovo, Russian Federation). As the control group, 187 healthy miners (control I) and 244 healthy male smokers living in the same area (Kuzbass), not working at the mine (control II) were recruited. In healthy donors, cancer had never been diagnosed. All of the subjects were Caucasian and without any psychiatric, hereditary or autoimmune disorders. Average work experience was 22.90 ± 10.07 years. A description of the studied groups is presented in Table 1. In the LC patient group, there was a significantly higher number of smokers than in the control group I (86% vs. 31%). Therefore, an additional control group (control II) was recruited, which included only heavy smokers (Pack-Years, PY > 25), without signs of oncopathology. In 81.25% of patients, non-small LC was diagnosed, of which 52.4% was at pTNM stages III and IV.

Recruitment was conducted according to the Declaration of Helsinki: all ethical principles of medical research were applied with modifications submitted in 2000. Before data and sample collection, all of the participants voluntarily provided informed consent. The study was approved by the Ethics Committee of The Federal Research Center of Coal and Coal Chemistry of Siberian Branch of the Russian Academy of Sciences.

Blood sampling was performed from ulnar veins with the usage of single-time vacuum systems «Vacutainer» by adding 0.25 mM EDTA-Na anticoagulant. Blood samples for genotyping were stored at −20 °C until use.

### 2.2. DNA Preparation and Genometype Analysis

DNA was extracted from peripheral blood with standard phenol-chloroform method. All blood cells were extracted and lysed and protein hydrolysis proteinase K (SibEnzyme, Novosibirsk, Russian Federation) was used. DNA was extracted by phenol and chloroform and precipitated by ethanol [38].

The polymorphisms of GSTM1 del genes were analysed by multiplex PCR. Each sample was amplified using the following pair of specific primers: F: 5′-GAACTCCCTGAAAAGCTAAAGC-3′; R: 5′-GTTGGGCTCAAATATACGGTGG-3′, designed in accordance with the fact that the lack of DNA matrix synthesis was matched to *GSTM1 del* deletion. The following primers were used for internal positive control, which was a fusible A/T-rich non-coding genomic fragment conventionally referred to as LTM (low temperature melting): F: 5′TGGGTGCTAGAGGTATAATCG3′; R: 5′TTAGAGGAAGCTGGGTAAGAG3′.

**Figure 1:**
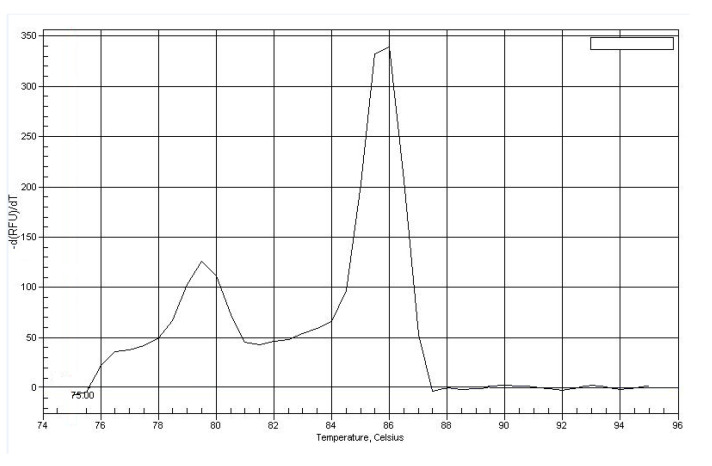
Melting curve plot for PCR products of *GSTM1* locus.

The total reaction volume was 25 μL. The mixture contained the following: 40–100 ng of DNA; 65 mMTris–HCl (pH 8.9); 0.05% Tween 20; 16 mM (NH_4_)_2_SO_4_; 2.4 mM MgCl_2_; 0.2 mMdNTP; 0.3 μM oligonucleotide primer solution; 0.8X SYBR Green I (SibDNA, Novosibirsk, Russian Federation) and 0.5 ed.ak. thermostableTaq-polymerase (SibDNA, Novosibirsk, Russian Federation). Amplification was performed using the thermocycleriCycler iQ5 (Bio-Rad, Hercules, CA, USA). The amplified fragment sizes in base pairs (bp) were as follows: GSTM1-229 and LTM-127. Results were interpreted after fluorescence accumulation plot analysis. Specificity was evaluated with a melting curve—the melting temperature for the *GSTM1* gene was 86.5 °C and that for LTM was 78.5 °C (Figure 1). The lack of fluorescent signal indicated homozygosity by this deletion (del). Heterozygotes by mutation were examined in the same group with individuals with normal genes (*n*).

Analysis of polymorphic variants of *APEX1* (*rs1130409*), *XPD* (*rs13181*), *NBS1* (*rs1805794*) genes was conducted by allele-specific PCR method using «SNP-express» kits (Lytech Research and Production Co., Moscow, Russian Federation) (Table 2). Amplification was performed by a thermocycler, applying a program included in the reagent kit manufacturer’s protocol. PCR products were analyzed by 3% agarose gel electrophoresis with ethidium bromide to visualize DNA fragments under ultraviolet light.

### 2.3. Statistical Analysis

For statistical data analysis, SNPStats (http://bioinfo.iconcologia.net/SNPstats, accessed on 28 January 2022) and STATISTICA 10.0 (StatSoft Inc., Tulsa, OK, USA) software were used. The frequency estimation of rare alleles was conducted using online resources (http://ihg.gsf.de/cgi-bin/hw/hwal.pl, accessed on 28 January 2022), accordance of genotype frequency distribution to the Hardy–Weinberg equilibrium (χ2), and also differences between comparing groups by allele and genotype frequencies. At *p* < 0.05, differences were defined as statically significant. Logistic regression analysis with odds ratio (OR) and 95% confidence interval (CI) calculation was adjusted for age and smoking status. To choose the most representative model, the smallest value of the Akaike informative criterion (AIC) was used.

For gene–gene interaction discovery, the multifactor dimensionality reduction (MDR) method was used [39]. It allows the verification of all possible models of SNP combinations. The contribution of every gene and/or their interaction is evaluated by the H parameter and represented in %. To conduct this analysis, MDR 3.2.0 (Computational Genetics Laboratory, Philadelphia, PA, USA) software was applied.

## 3. Results

**Figure 2:**
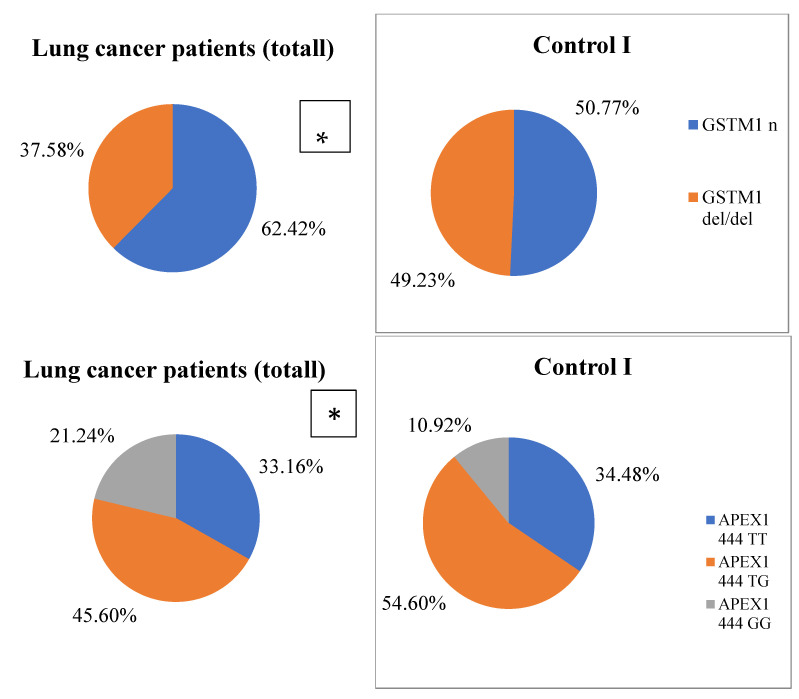

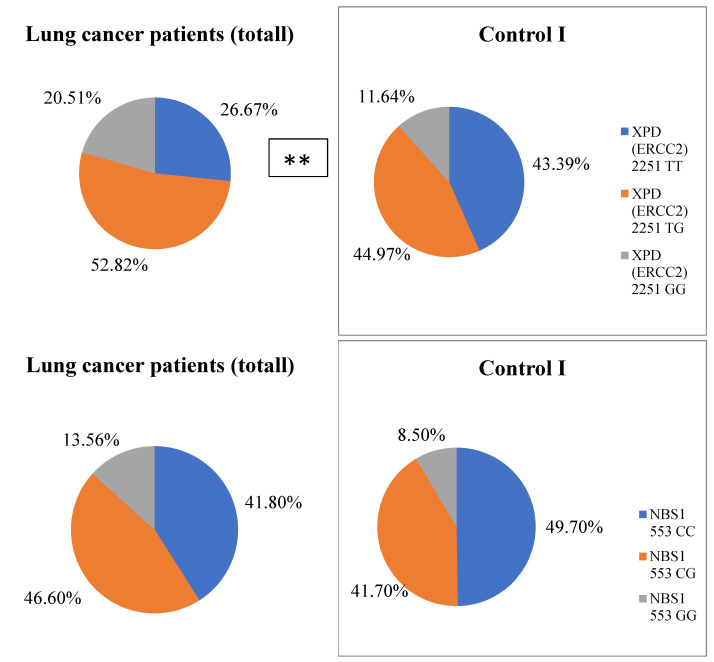
Genotype frequency distribution in the studied groups. Differences between LC patients and Control I * *p* = 0.02; ** *p* = 0.001.

**Figure 3:**
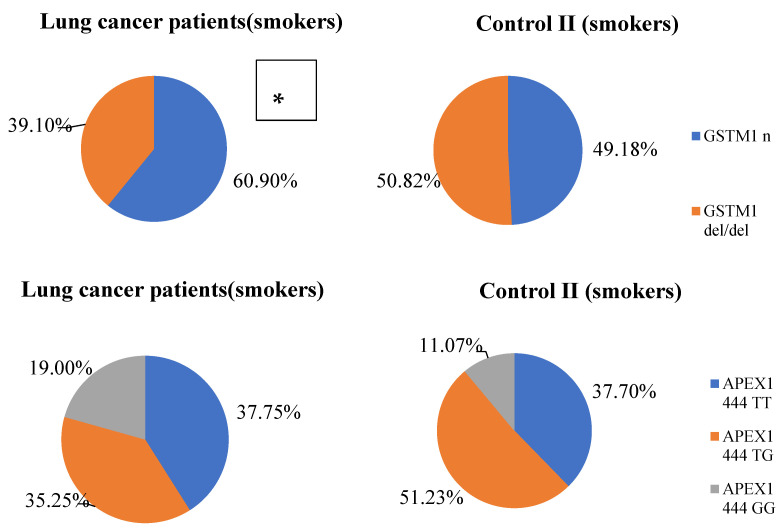

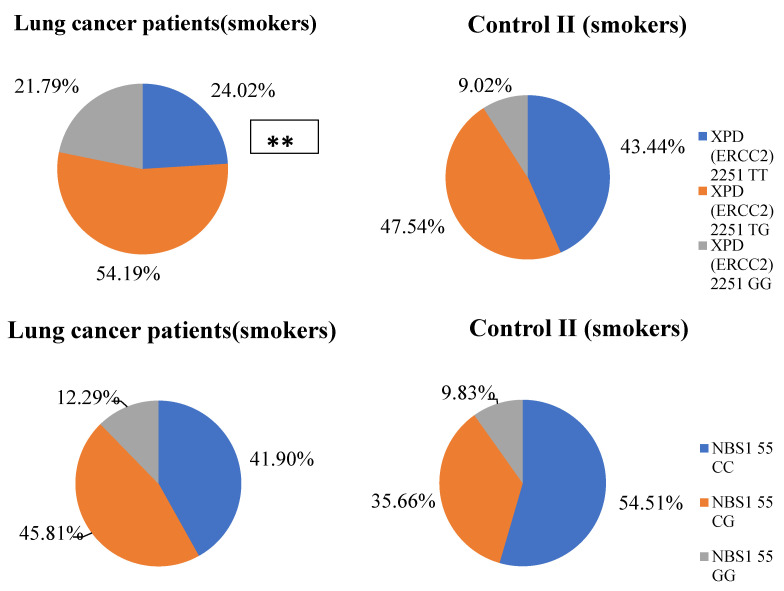
Genotype frequency distribution in smokers. * Differences between LC patients and Control II * *p* = 0.02; ** *p* = 0.00002.

Analysis of biotransformation and DNA repair enzymes gene polymorphsms was performed in a cohort of coal miners with LC and in healthy coal miners of the Kemerovo region. Results are presented in Figure 2 and Figure 3. The distribution of genotypes in the studied groups corresponded to the Hardy–Weinberg equilibrium and to parameters found in European populations [40,41]. No statistically significant differences in LC patients group related to disease stages (TNM1 and TNM2 vs. TNM3 and TNM4) and tumor localization were detected. A statistically significant difference in *GSTM1* (del) and *XPD 13181* T > G, alleles and genotype frequency distribution between healthy individuals and LC patients was identified.

By regression analysis, adjusted for age and smoking, significant LC risk decreases with deletion of *GSTM1* (ORadj = 0.46, CI 95%: 0.25–0.83; Padj = 0.009). Increased risk of LC development was identified for *XPD 2251* T > G polymorphic locus in coal miners patients (dominant model: ORadj = 1.75; CI 95%: 1.05–2.92; Padj = 0.033; additive model: ORadj = 1.55; CI 95%: 1.08–2.22; Padj = 0.016). A significant association was detected between *APEX1 444* T > G locus and LC risk in coal miners in a recessive model of heritage (adjusted for age and smoking: ORadj = 2.65; CI 95%: 1.29–5.43; Padj = 0.006).

At the next stage of the study, we analyzed the distributions of alleles and genotypes only in the groups of smokers (patients with lung cancer and healthy men). Results are presented in Figure 3. A statistically significant association of the deletion genotype of the *GSTM1* gene with a decrease in the risk of developing LC in miners was found (ORadj = 0.59, CI 95%: 0.36–0.98; Padj = 0.04). Using regression analysis, adjusted for age, a statistically significant association for the risk of developing LC in miners was found with the *APEX1 444* T > G locus in the recessive inheritance model (ORadj = 1.87; CI 95%: 1.01–3.48; Padj = 0.047), and with the polymorphic locus *XPD 2251* T > G in the additive inheritance model (ORadj = 2.25; CI 95%: 1.59–3.19; Padj = 0.0001).

**Figure 4:**
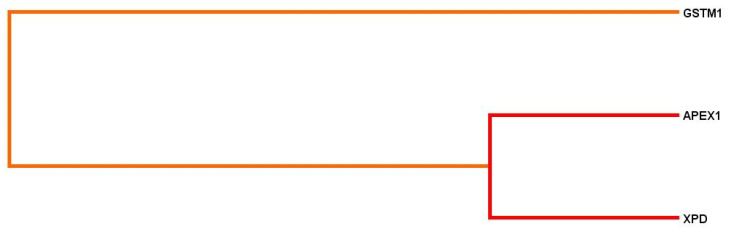
Dendrogram of gene–gene interactions during lung cancer development. Short lines point to a strong level of gene loci interactions; long lines weak association; red and orange lines correspond to synergism (strong effects between loci).

Using the multifactor dimensionality reduction (MDR) method, the statistically significant 3-loci model (*p* = 0.00001) of gene–gene interactions was found. It was characterized by a good precision (precision significant test *p* = 0.703 at maximum 1.0) and maximal statistical value of reproducibility (cross-validation consistency: 10/10). The most significant contribution to disease development in the presented model was defined for *APEX1* (*rs1130409* T > G) (H = 1.20%) and *XPD* (*rs13181* T > G) (H = 0.99%) loci (Figure 4) (for 3- loci model: OR = 3.0905; CI 95%: 1.81–5.29).

Cluster analysis demonstrated a strong association and synergism between *APEX1* (*rs1130409* T > G), *XPD* (*rs13181* T > G) and *GSTM1* (del) loci. (Figure 4).

**Figure 5:**
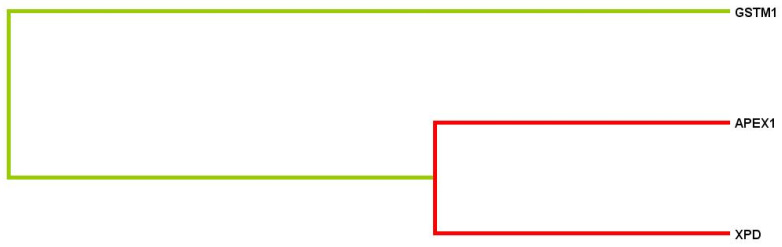
Dendrogram of gene–gene interactions during LC development. Short lines point to a strong level of gene loci interactions; long lines indicate weak association; red lines correspond to synergism (strong effects between loci); and green lines demonstrate double effects between loci.

When only smokers were compared (miners with lung cancer and control II), a similar pattern of intergenic interactions was observed. The most significant contribution to disease development in the presented model was defined for *APEX1* (*rs1130409* T > G) (H = 1.46%) and *XPD* (*rs13181* T > G) (H = 1.41%) loci (Figure 5) (for the three-loci model in the group of smokers: OR = 2.79; CI 95%: 1.73–4.52).

Cluster analysis demonstrated a strong association and synergism between *APEX1* (*rs1130409* T > G), *XPD* (*rs13181* T > G) and double effects with *GSTM1* (del) loci. (Figure 5).

## 4. Discussion

Cancer risk is a complex interplay between hereditary genetic predispositions, living and working environmental exposure, age and sex. The potential of environmental carcinogens for cancer development is determined by the functional activity of biotransformation enzymes and DNA repair capacity.

The link between the *GSTM1* polymorphism and LC risk has frequently been studied, but the obtained results are still controversial. The results of the current study are in accordance with those presented by Shilova et al. (2008), who showed that the deletion of the *GSTM1* gene decreased susceptibility to larynx cancer [42]. Yadav D.S. (2010) also noticed the protective effects of *GSTM1* del genotype with regard to LC risk in North-East India residents [43]. However, some studies gave the opposite results, where the deletion of *GSTM1* was associated with an enhanced risk of non-small LC in Mongols and Chinese people, lung adenocarcinoma in North Indian residents and small-cell LC in South Indian inhabitants [23,24,44]. The significance of the *GSTM1* del genotype in Pakistani, Turkish and Belarussian populations with respect to lung malignancy was not confirmed in previous studies [45,46,47].

A coal miner’s working environment implies complex radiochemical exposure to radon, particles of different sizes and carcinogenic chemical substances such as PAHs, all of which have been associated with LC [48,49,50]. A large number of coal miners are smokers [51], and in our study, among the LC patients, there were significantly more subjects who were smokers than in the control group I. Therefore, an additional comparison group was formed, consisting only of heavy smokers (control II). The PAH epoxides formed during tobacco smoking are substrates for the GSTM1 enzyme [52]. It was shown that the deletional genotype of *GSTM1* in individuals causes decreased levels of 8-oxoguanine, which can be formed by the mutagenic effects of the PAHs benzo(a)pyrene and benz(a)anthracene [53]. The formation of free 8-oxoguanine is accompanied by apurinic site (AP-site) occurrence in DNA, and the APEX1 enzyme promotes their recognition and elimination. Lung-deposited silica or coal dust inhibits the induction of cytochrome P4501A1 by polycyclic aromatic hydrocarbons. It has been hypothesized that the resulting lower cytochrome activity might to some extent counteract the carcinogenic effects of tobacco smoke by limiting the metabolism of PAHs in tobacco smoke into carcinogenic metabolites [54].

Apurinic/apirimidinic endonuclase is an essential enzyme that participates in the base excision repair (BER) pathway. The *APEX1* (*rs1130409* T > G) polymorphism leads to Asp on Glu substitution in the 148 codon, thereby decreasing the ability of this protein to interact with another enzymes and reducing DNA repair efficiency [55]. Our analysis detected a statistically significant association of the *APEX1 G444G* genotype with LC (in a recessive inheritance model), which is consistent with the results of several studies [25,26,56]. In particular, the specific allele of *APEX1* was linked to a high risk of LC in Chinese coal mine workers who were exposed to high concentrations of PAHs [56]. In Chen et al. (2013), a direct association between *APEX1* (rs1130409 T > G) polymorphism and LC risk was not obtained, but it was supposed that the smoking carriers of this minor allele had an increased predisposition for the disease [57].

The *NBS1* gene (Nijmegen rupture syndrome) encodes nibrin, which plays an important role in double-strand break repair and participates in signal transduction and telomere structure stabilization [58]. C on G transversion, which causes substitution of glutamate to glutamine (Glu185Gln, rs1805794), has been studied in the context of LC risk, but the obtained results are quite controversial [27,59,60]. In the current study, it was shown that the NBS1 GG genotype is not associated with increased LC risk in coal miners. In the Chinese population, the *NBS1* minor allele had enhanced risk for LC [60], but in Taiwanese people, the *NBS1 G553G* variant had a similar association only in male smokers [27]. In a group of Caucasians (Norwegian general population), such an association with the risk of developing non-small cell LC was also detected [59].

The Xeroderma Pigmentosum Complementary group D (XPD) is involved in the nucleotide excision repair (NER) pathway. *XPD* gene polymorphism at position 751 in exon 23 (rs13181), resulting in a lysine-to-glutamine transition, may alter the interactions of different proteins, reduce the activity of TFIIH complexes and modulate genetic susceptibility to cancer. The analysis pointed to a strong linkage between high LC risk in coal miners and the *XPD* (*rs13181* T > G) polymorphism. The variant allele of the *XPD* gene decreases DNA repair efficiency, which has been confirmed by results presented previously [61]. An association of this polymorphic variant with LC in smoking and non-smoking European and Asian residents was also established [26,59,62,63,64,65]. A product of the *XPD* gene can recover all DNA aberrations caused by PAH activity. This fact proves the existence of a strong association and synergism between the *APEX1* (*rs1130409* T > G) and *XPD* (*rs13181* T > G) loci, as obtained in our cluster analysis.

The results of the study are partially consistent with the data of the study of residents of the Kemerovo region carried out earlier [37]. In residents who did not work in the coal mining industry, only *XPD* (*rs13181* T > G) was shown to increase LC risk. The *APEX1* (*rs1130409* T > G) variant promoted increased risk only in smoking patients. In a cohort of Kemerovo region residents, the deletional genotype of the *GSTM1* gene was not associated with LC development in contrast to coal miners. Along with that, the performed MDR method demonstrated another model of gene-gene interactions of LC risk formation in patients who had no exsposure associated with the coal mining industry in contrast to coal miners. This model included the interaction of *XRCC1* (*rs25487*), *NBS1* (*rs1805794*), *hOGG1* (*rs1052133*) and *XPG* (*rs17655*) loci in Kemerovo region residents versus *APEX1* (*rs1130409* T > G), *XPD* (*rs13181* T > G) and *GSTM1* (del) in coal miners with LC [37]. It could be suggested that these dissimilarities may have been caused by different qualitative and quantitative characteristics of pollutants that can activate different mechanisms in coal mine workers and the general population.

## 5. Conclusions

In conclusion, the obtained results in the current study demonstrated an association between a combination of polymorphic variants in the tested genes and LC risk in coal miners. Disease progression is determined by the interactions of genes involved in antioxidant activity (*GSTM1*) and DNA repair (*APEX1*, *XPD*). This group of biomarkers may be used in future as a tool for identifying susceptible subjects with higher cancer risk in order to create preventive measures.

## Figures and Tables

**Table 1:** Descriptive analysis of the study participants.

Parameters		Lung Cancer Patients	Control I	Control II
Average age, years (Mean ± S.D.)		59.04 ± 6.55	58.98 ± 4.99	57.83 ± 6.09
Number of subjects (N)		208	187	244
Smoking status	Smokers	179	58	244
	Pack-Years, PY *	32.5	25.0	31.6
	Non-smokers	29	129	-
Cancer type	Small cell lung cancer	39		
	Non-small cell lung cancer	169		
pTNM	0, I, II	99		
	III, IV	109		

* PY = $\frac{N*n}{20}$; *N*—the number of cigarettes smoked per day, *n* is the smoking experience, years, 20 is the number of cigarettes in one pack.

**Table 2:** Characteristics of the loci and primers used for analysis by allele-specific PCR.

Gene	Polymorphic Loci (Ref SNP)	Allele	Primers (5′→3′)
*APEX1*	444 T > G (rs1130409)	T, G	F: 5′-ATTGAGGTCTCCACACAGCACA-3′ R: 5′-AATTCTGTTTCATTTCTATAGGCGAG-3′
*XPD* (*ERCC2*)	2251 T > G (rs13181)	T, G	F: 5′-TCAAACATCCTGTCCCTACT-3′ R: 5′-CTGCCGATTAAAGGCTGTGGA-3′
*NBS1*	535 C > G (rs1805794)	C, G	F: 5′-GCAGTGACCAAAGACCGACTTCTA-3′ R: 5′- TGAGGTTACCTCAGTGCCATTTACT-3′
